# Assessment of Visual Quality Improvement as a Result of Spectacle Personalization

**DOI:** 10.3390/life13081707

**Published:** 2023-08-08

**Authors:** Fruzsina Benyó, Lilla István, Huba Kiss, Andrea Gyenes, Gábor Erdei, Éva Juhász, Natalia Vlasak, Claudia Unger, Tamás Andorfi, Kata Réz, Illés Kovács, Zoltán Zsolt Nagy

**Affiliations:** 1 Department of Ophthalmology, Semmelweis University, 1085 Budapest, Hungary; benyo.fruzsina@med.semmelweis-univ.hu (F.B.); istvan.lilla@med.semmelweis-univ.hu (L.I.); kisshuba@googlemail.com (H.K.); gyenes.andrea@med.semmelweis-univ.hu (A.G.); juhasz.eva@med.semmelweis-univ.hu (É.J.); nagy.zoltan.zsolt@med.semmelweis-univ.hu (Z.Z.N.); 2 Department of Atomic Physics, Institute of Physics, Budapest University of Technology and Economics, 1111 Budapest, Hungary; 3 Hoya Vision Care, 1043NX Amsterdam, The Netherlands; natalia.vlasak@hoya.com (N.V.); claudia.unger@hoya.com (C.U.); 4 Department of Clinical Ophthalmology, Faculty of Health Sciences, Semmelweis University, 1088 Budapest, Hungary; andorfitamas@gmail.com (T.A.); rez.kata@semmelweis.hu (K.R.)

**Keywords:** personalized spectacle, visual quality, progressive addition lens

## Abstract

Personalized spectacles customized according to an individual’s facial anatomy were developed to provide enhanced visual performance and overall comfort when compared to standard spectacles. In this comparative crossover trial, each subject was randomly assigned to wear either personalized spectacles or standard spectacles for two weeks and then tried the second pair for another two weeks. Visual acuity and reading speed were measured, and visual quality and comfort were assessed using specific questionnaires. The correlation of the wearing parameters with the subjects’ satisfaction was calculated. According to our results, the subjects wearing personalized glasses reported significantly less experience of swaying and significantly higher overall satisfaction compared to those wearing the control spectacles. At the end of the study, 62% of subjects preferred the personalized spectacles, and visual quality was the primary reason for their spectacle preference followed by wearing comfort. The difference from the ideal cornea–vertex distance was significantly lower when wearing the personalized spectacles compared to the control frames. In addition, the absolute value of the difference from the ideal cornea–vertex distance was significantly correlated with patient satisfaction. These results suggest that personalized spectacles, customized according to an individual’s facial anatomy for the ideal wearing parameters, result in both visual and comfort advantages for wearers.

## 1. Introduction

Proper corrective glasses play a vital role in our modern lifestyle. With the increasing reliance on digital devices and the prevalence of near work activities, such as reading, writing, and using computers, our eyes are constantly under strain. Corrective glasses, tailored to our specific visual needs, help alleviate the discomfort and potential eye strain caused by refractive errors and presbyopia during quick adaptation to different distances when using computers, tablets, and smartphones [1]. Corrective lenses include distance glasses, reading glasses, bifocals, and progressive addition lenses, [2] the latter of which provide the desired additional power by continuously increasing the dioptric power between the distance and near zones of the lens [3,4,5,6,7]. It is known that some presbyopes wearing progressive addition lenses experience moderate-to-severe visual [8] symptoms, such as blurred vision, headaches, peripheral visual field distortion, imbalance, and even nausea [9], which eventually lead to the discontinuation of progressive addition lenses. The reason why some presbyopes find it so difficult to adapt to progressive addition lenses, while others find them easy to wear, is not fully understood [10,11]. The spectacle lens industry has been working for many decades to improve the design of spectacle lenses in order to meet this increasing demand for comfortable vision [12,13]. An early type of progressive addition lens was produced based on standardized wearing parameters and was a symmetrical design that showed typical side effects, such as headaches, a narrowed field of vision, a swimming/rolling sensation, discomfort, or feeling unsafe on stairs [14]. Initially, a long period of adaptation and high motivation was required from the wearers [15,16]. Even after a long period of adaptation, some wearers rejected the spectacles because they did not adapt. For these subjects, the inconvenience and lack of visual comfort and performance outweighed the benefits of perfect vision at all distances [17].

As shown previously, high visual performance is strongly linked to how a lens is positioned in front of the eyes [18], and thus, theoretically, individualized spectacles could provide better acceptance by both monofocal and multifocal spectacle wearers because of the improved visual comfort and performance offered [19]. This performance enhancement is currently limited by standardized spectacle frames as they only allow for the adaptation to anatomical and morphological facial requirements in a certain range. In the past few years, a new generation of mono- and multifocal lens design has been developed considering, for the first time, multiple individual spectacle frame parameters, such as wearers’ pantoscopic angle (WPA), frame face form angle (FFA), frame–cornea distance (FCD), and frame-related centration data. Initially manually determined, the so-called video-centration measurement systems soon made it possible to measure the frame-related parameters directly on the worn frame more precisely. At the same time, to achieve the optimal lens position for the individual, the frame should also be individually tailored.

The purpose of this study is to assess the visual performance and wearing comfort of personalized spectacles produced by taking into account the individual facial parameters of a wearer compared to data obtained from wearing spectacles using a standard pre-produced frame.

## 2. Materials and Methods

This comparative study was conducted at the Department of Ophthalmology, Semmelweis University, Budapest, Hungary. The study followed the tenets of the Declaration of Helsinki and was approved by the Ethics Committee of Semmelweis University (29819/AOSZE/2018) before commencement. After the protocol had been fully explained, all subjects provided written informed consent to participate in the study spontaneously and for free and gave permission to collect and examine their personal and optometric data. 

### 2.1. Eligibility Criteria

Altogether, 60 subjects were enrolled in this study. The subjects were experienced wearers wearing a single vision/progressive addition lens design during the previous six months with the best-corrected monocular visual acuity for distance and near ≥ 1.0 decimal. The participants required a new eyeglass prescription and had normal binocular vision and ametropia between −6.00–+6.00 spherical diopter and a cylindrical diopter ≤ 2.50D. The difference in the power (spherical equivalent) between the eyes was ≤2.00 D. A detailed description of the inclusion and exclusion criteria can be found in Table A1.

In total, 30 subjects wore a single vision lens design (SV group) and 30 subjects were prescribed a progressive addition lens design (PAL group). 

### 2.2. Study Design

Each subject was randomly assigned to wear either the personalized spectacles or the standard spectacles for a period of two weeks. The subjects then tried the second pair (crossover) for another period of two weeks. The spectacles were dispensed in an unmarked box. During the study, the investigators who performed the measurements and the subjects were blind to which spectacles were worn by which subjects, so it was impossible to differentiate between the personalized and standard spectacles.

### 2.3. Study Devices

Two types of spectacles were worn during the study: the personalized spectacles and the control spectacles, which were visually indistinguishable; both had identical material, color, and shape. All personalized spectacles were produced using HOYA’s patented YUNIKU technology.

### 2.4. Personalized (3D Tailored) Spectacles

**Figure 1:**
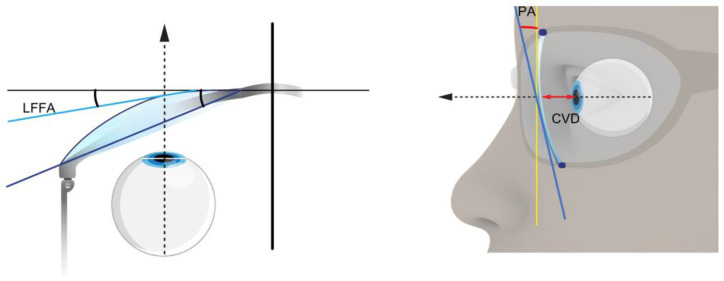
Individual lens-wearing parameters: CVD: cornea–vertex distance; PA: pantoscopic angle; and LFFA: lens face form angle.

YUNIKU spectacles consist of a personalized frame, produced on demand for each subject, and standard lenses in the ideal position. Before producing the YUNIKU spectacles, the subjects’ faces were scanned by a YUNIKU scanner, and 3D images of their faces were rendered by YUNIKU software. Prescription and satisfaction levels with previous spectacles, functional requirements according to the spectacles’ usage, and the facial reference points of each subject were collected by the YUNIKU software. The algorithm used these data to calculate the ideal position of the lens. Taking the lens’ position into account, a virtual frame model is adjusted to the calculated lens parameters and to the morphology of the face. The frame is produced on demand for each subject using laser sintering technology, which is an additive manufacturing process that uses a high-powered laser to fuse powdered materials together to create three-dimensional objects. This technology offers several advantages over traditional manufacturing methods of spectacle frames as it allows for the creation of individual geometries that may be difficult or impossible to achieve with traditional manufacturing techniques. The lens position is designed corresponding to the calculated individual lens-wearing parameters (Figure 1). Both the YUNIKU single vision lens position and the YUNIKU progressive addition lens position were determined in a 3D design space.

### 2.5. Control Standard Spectacles

**Figure 2:**
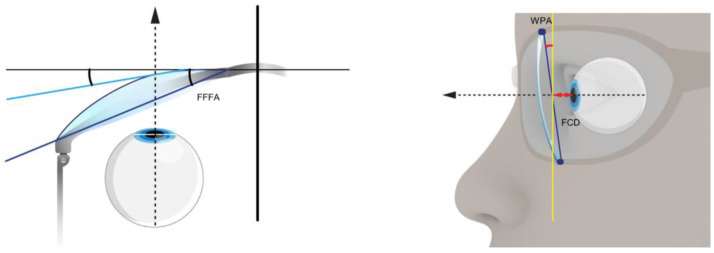
Frame-related wearing parameters: FCD: frame–cornea distance; WPA: wearers’ pantoscopic angle; and FFFA: frame face form angle.

The frame of the control spectacles is pre-produced in one standard fixed size. The frame size according to the boxing system and bridge is adapted to facial needs individually in steps of 1 mm. The frame has the same look, material, color, and shape as the individual YUNIKU frame. The same aspherical single vision lens design and progressive addition lens design were used for both the control spectacles and the YUNIKU frames. The frame-related parameters were measured by using a video-centration system called visuReal portable (Figure 2). 

Subjects with inadequate fitting of the control frame according to the standard care of prescribing spectacles by a trained optometrist were excluded from the study. All YUNIKU and control lenses were verified by the binocular eye model [20] to ensure seamless matching with the visual system, resulting in supreme depth perception. During the production process, each progressive addition lens is corrected by binocular harmonization technology (BHT) leading to better intermediate and near vision. BHT recalculates and adjusts the progressive power distribution according to the actual used positions on each lens so that both eyes will experience the same accommodation support. The main differences between the construction of personalized spectacles and control standard spectacles are summarized in Table 1. 

### 2.6. Outcomes

The primary outcome of the study was to identify a preference for either the personalized spectacles or the control standard spectacles at the end of the study. During the study, visual quality and spectacle comfort were evaluated by specific questionnaires assessing patients’ first impression (Table A2), subject satisfaction (Table A3), and final comparison (Table A4) with responses to questions scored 0–5 or 1–10 as indicated. Secondary outcomes presented in this article include the evaluation of visual performance by the 25-item Visual Function Questionnaire (VFQ-25) and by measuring the reading speed by using a standard Radner test. The Radner Reading Charts consist of standardized “sentence optotypes” that logarithmically progress in print size and were designed for clinical and research use. This test provides a number of different reading parameters from a single examination in patients with normal-to-low vision, and its reliability and validity have already been demonstrated [21,22].

### 2.7. Statistical Analysis

A statistical analysis was performed by using SPSS software (version 23.0, IBM, Armonk, NY, USA). The sample size was determined a priori by statistical power calculation (power 0.80; *p* = 0.05), and the minimum number of patients to enroll in this study was calculated to be 58. The Shapiro–Wilk W-test was used to test the normality of the data. Due to the non-normal distribution of the data, the Wilcoxon signed rank test for the dependent samples was used to analyze the differences between the scores of different subsets of questionnaires obtained from the two different spectacles. The Chi-square test was used to test relationships between categorical variables. The binomial test with confidence intervals was used to analyze the preferences of frame selection. Principal component analysis using Varimax rotation was conducted to examine the factor structure of the final comparison questionnaire. Principal component analysis allows a large number of variables to be condensed into relatively few new variables, or principal components, that give the most information about the data. These principal components were then analyzed to determine which ones correlated with the spectacle preference. In all statistical analyses, a *p*-value of less than 0.05 was considered to be statistically significant.

## 3. Results

None of the 60 enrolled subjects (female: 37; male: 23) was excluded from the study, and all subjects completed all visits. All patients had a best-corrected distance visual acuity and best-corrected near visual acuity of 1.0 (i.e., 0.0 logMAR) or better. The subjects’ mean sphere refraction error was −1.58 ± 2.51 diopter in the SV group and −0.34 ± 2.55 diopter in the PAL group (*p* = 0.03). The cylindrical error was −0.49 ± 0.66 diopter in the SV group and −0.45 ± 0.41 diopter in the PAL group (*p* = 0.86). Patients in the PAL group required an average of 2.17 ± 0.43 addition in their progressive addition lenses. The fitting properties of the spectacles are presented in Table 2.

### 3.1. Evaluation of Visual Functions and Reading Speed

There was no difference in the VFQ-25 scores when wearing the personalized spectacles compared to wearing the control spectacles (93.29 ± 6.01 vs. 93.27 ± 5.87; *p* = 0.82). However, the subjects showed significantly higher reading speeds (less time to read the Radner chart) when wearing the personalized spectacles in comparison to when wearing the control spectacles (5.73 ± 1.05 s vs. 5.79 ± 1.01 s; *p* = 0.04).

### 3.2. First Impression Questionnaire

According to the results of the first impression questionnaire, there was no significant difference between the personalized and control spectacles in any items related to visual quality or comfort provided by the spectacles (Table A5).

### 3.3. Subject Satisfaction Questionnaire

Overall satisfaction with each pair was assessed using the satisfaction questionnaire, which was completed after 14 days of wearing the new spectacles. Significantly higher scores were reported in distance (4.65 ± 0.68 vs. 4.25 ± 1.08; *p* = 0.009) and near vision (4.48 ± 0.95 vs. 4.13 ± 1.17; *p* = 0.01) by subjects wearing the personalized spectacles (Table A3). In addition, the subjects reported significantly less experience of swaying (4.40 ± 0.92 vs. 4.00 ± 1.18; *p* = 0.02) and significantly higher overall satisfaction (4.13 ± 0.91 vs. 3.73 ± 1.15; *p* = 0.03) when wearing the personalized spectacles (Table A6).

### 3.4. Final Comparison Questionnaire

There was no statistically significant difference in the answer scores in the final comparison questionnaire, which was completed during the last visit after the subjects tried both spectacles. As a primary outcome of the study, 37 (62%) out of 60 subjects chose the personalized spectacles as the ones they would keep. This preference towards the personalized spectacles was statistically significant (*p* = 0.04; Table 3). However, there was no statistically significant preference for the personalized spectacles when analyzing the SV and PAL groups separately (*p* > 0.05). 

**Figure 3:**
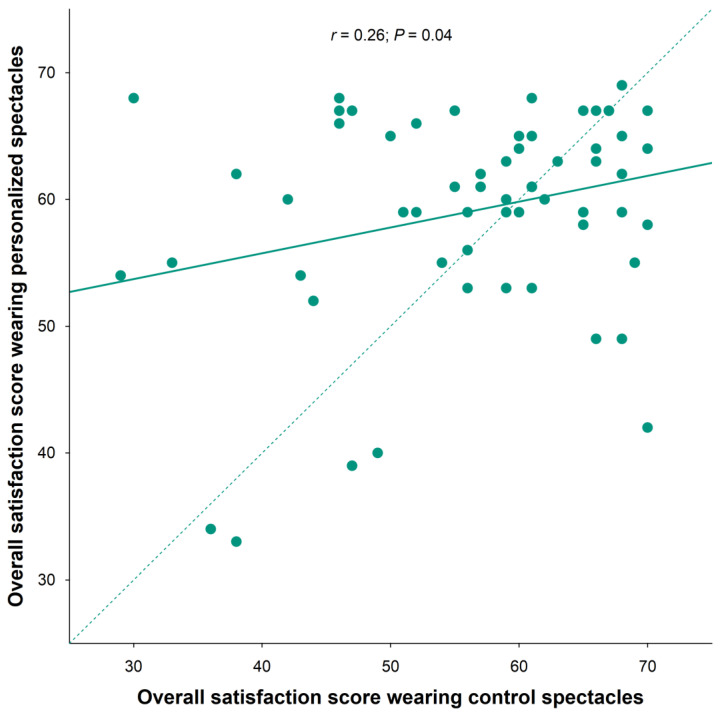
Correlation of overall satisfaction scores between the two study spectacles. Each circle represents a specific subject.

When analyzing the overall satisfaction after wearing both of the studied spectacles, the subjects tended to be more satisfied with the personalized spectacles (Figure 3).

The principal component analysis identified two factors that accounted for 75.93% of the variance in the final comparison questionnaire scores. The first component (visual quality) explained 54.45% of the variance and was primarily responsible for the spectacle preference, while the second component (spectacle comfort) only accounted for 21.48% of the variance in scoring and had a secondary role in spectacle preference.

**Figure 4:**
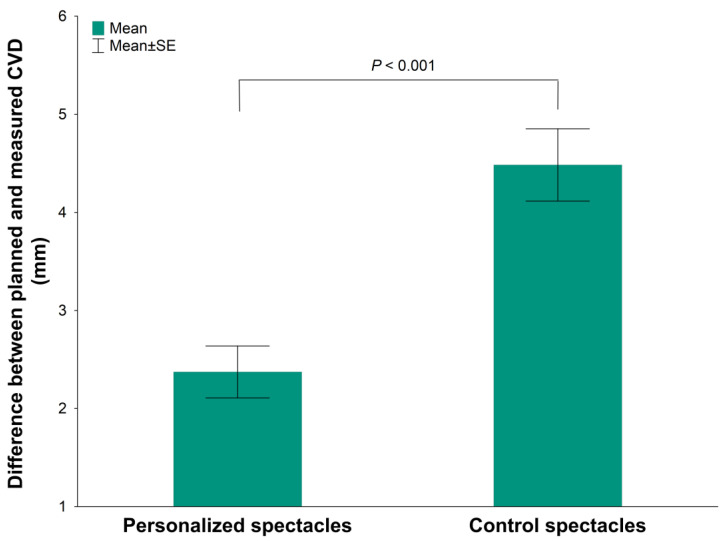
Absolute value of the difference in the measured CVD from the planned values when wearing the personalized and control spectacles. Data: mean ± standard error.

Finally, we found a statistically significant difference between the personalized and control spectacles in terms of deviation from the ideal cornea–vertex distance values (12.5 mm), which is the design target of the spectacle lenses, in order to keep the spectacle magnification around unity. This value was set in the trial frame during refraction. In the case of the personalized frames, the mean difference was much lower than for the control frames (2.37 ± 2.05 mm vs. 4.48 ± 2.85 mm; *p* = 0.001, Figure 4), which clearly shows the effectiveness of frame customization.

**Figure 5:**
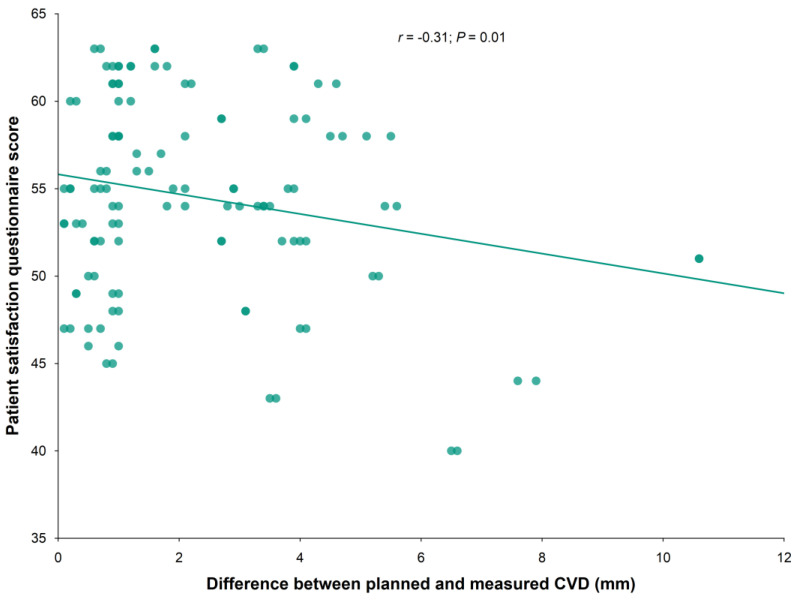
Correlation between deviation from the ideal CVD and patient satisfaction.

In addition, the correlation analysis showed that the absolute value of the difference (in mm) from the ideal cornea–vertex distance value for the personalized spectacles was significantly correlated with patient satisfaction (*p* = 0.01). The correlation coefficient resulted in being r = −0.31, implying that a decreasing position error increases patient satisfaction (Figure 5).

Regarding the other parameters, the lens face form angle’s differences from the ideal value (0°) did not show a similar effect on patient satisfaction (*p* = 0.59), while the pantoscopic angle had no specific ideal value at all. The ideal LFFA was the design target of the spectacle lenses, defined as LFFA = 0°, since the human eye looks straight forward on average; thus, its optical axis can be considered as lying in the vertical plane of the head. PA is different for each facial structure and visual situation (reading, watching TV, driving, etc.); this is why an ideal position cannot be defined.

## 4. Discussion

In this clinical study, we have demonstrated that although there was no immediate benefit after putting on personalized spectacles when wearing them for a long time, the subjects were more satisfied with distance and near vision, reported less swaying, and were more satisfied in general compared to subjects wearing the control spectacles. After completing this crossover comparative study, we have shown that a significantly larger proportion of subjects preferred the personalized spectacles over the control spectacles, suggesting that improved visual performance and overall comfort while wearing the personalized spectacles is noticeable even when the best-corrected visual acuity is similar with these two spectacles. By using principal component analysis of the patient satisfaction questionnaires, we have shown that visual quality was the primary component that correlated with the preference to choose personalized spectacles. Spectacle comfort, the second principal component, was uncorrelated with visual quality and was found not to be of major importance in spectacle preference. Although the difference between the personalized and control spectacles in terms of subjective experiences was relatively small, patient satisfaction with the personalized spectacles was dependent on deviation from the ideal cornea–vertex distance, thus demonstrating the clinical relevance of proper lens positioning to deliver the best visual quality. The deviation of the actual cornea–vertex distance from the ideal cornea–vertex distance was significantly lower for the personalized frames. The level of satisfaction was proportional to this deviation, as the closer the cornea–vertex distance was to the optimal value, the higher the level of satisfaction reported by the participants. An interesting observation was that people who gave a lower score for comfort when wearing standard glasses had a greater improvement in satisfaction scores when wearing customized glasses. Although the reasons for this finding were not investigated in this study, it may be due to the more unusual face form of these people, which makes it more difficult to fit standard spectacles correctly.

The results of this study are particularly important for progressive addition lens wearers, who usually experience some discomfort symptoms related to the progressive addition lens design [23]. Since many factors may lead to discomfort [24], such as the effect of a small reading area on depth perception and contrast sensitivity [25,26] or a narrow corridor due to the large amounts of astigmatism at the edges, eye care professionals do not have a standardized technique to determine which patients will likely adapt to progressive addition lenses and which patients will have a difficult time. The design and width of the progressive addition lens channel can vary depending on the specific lens manufacturer and the wearer’s prescription needs. Some progressive addition lenses may have wider channels, providing a larger area for near-vision tasks, while others may have narrower channels that prioritize distance vision. It is also known that ear position has a significant effect on pantoscopic angle, resulting in an unwanted tilt of spectacle lenses leading to visual disturbances or a long period of adaptation to progressive addition lenses [25,26]. The pantoscopic angle is defined not only by the position of the wearers’ ears on their heads in relation to their eyes but also by the predefined inclination of the spectacle frame, which can be slightly adjusted. In order to compensate for a pantoscopic angle that is not ideal, the progressive lens design is converted to that angle. However, even though the technology for modern lens design is much more enhanced than 60 years ago, there are still cases of non-adaptation.

Wearing the correct progressive addition lenses is particularly important for people working on computers as screen time, including smartphone use, has increased significantly in Western countries over the past decade. In 2020, adults in the United States spent an average of 13 h a day interacting with media, including screen time on computers, smartphones, tablets and TVs. This is a significant increase from 10 years ago, when average daily screen time was around 7 h. Advances in technology have also increased the resolution of such devices: smartphones with more than 300 dpi and UHD/4K desktop monitors have been with us for more than a decade, both of which challenge the eye’s resolution capacity. Improved visual quality is therefore increasingly important, as it can have a direct impact on the effectiveness of computer work. When people have clear and sharp vision, they can read text, view images and interpret visual data with greater accuracy and efficiency. This reduces the time spent deciphering unclear or blurred information, leading to improved productivity and task completion. In addition, improved visual quality reduces eye strain and fatigue, allowing people to work comfortably for longer periods of time without experiencing visual discomfort. It is also important to note that inappropriate spectacle correction can aggravate dry eye symptoms, leading to increased discomfort and visual disturbance. 

The best possible position of corrective spectacles on the subject’s face is a prerequisite for quick adaptation and the best optical quality provided by the spectacles [27]. The fitting of a frame by an optometrist is a collaborative process that involves understanding the individual’s facial anatomy, prescription requirements, and personal preferences. During a frame fitting, the optometrist considers various factors to determine the most suitable frame for the individual. They take into account facial dimensions, such as interpupillary distance and facial width, to ensure proper alignment of the lenses with the individual’s eyes. The optometrist also considers the shape of the individual’s face and features to recommend frames that complement their facial structure and personal style. Additionally, the optometrist assesses the position of the ears and the shape of the nose to ensure a comfortable and secure fit. They make necessary adjustments to the frame, such as temple length and nose pad positioning, to customize the fit for the individual. The optometrist also considers the individual’s prescription needs, ensuring that the frame can accommodate the required lens thickness and curvature. By taking into account these factors, the optometrist ensures that the selected frame provides the best possible visual experience and meets the individual’s unique needs. However, new technologies allow many more options for personalization as biometric data can be captured quickly and with high resolution, using a 3D scanner and personalized manufacturing production methods, such as the laser sintering process [28,29,30]. Instead of taking standardized spectacle frames with their limitations as given and accepting the associated constraints in visual performance and wearing comfort, new technologies can be used in such a way that allows personalized spectacles to optimize the position of the lens in front of the eye, providing a higher level of visual performance and wearing comfort. 

The personalized spectacles, known as YUNIKU, are considered a completely new approach to fully personalized spectacles, as the YUNIKU software calculates the ideal position of the lens in relation to the eyes and then designs the frame based on those unique parameters. Laser sintering technology allows tailoring of the frame to optimize visual, aesthetic, and comfort performance, and lens design does not need to be reverse-adjusted based on frame-related parameters. According to the results of this clinical study, this level of spectacle personalization provides slightly better visual performance and satisfaction than the control spectacles. In this study, we have shown that reading speed was also slightly better when wearing personalized spectacles. Although the difference was minimal, faster reading might be attributed to the less compromised visual quality due to more optimal lens tilt in the personalized spectacles. The significance of this finding is that previous research on people working at a computer screen has shown that increasing reading speed can lead to improved comprehension, increased productivity and reduced eye strain. As unwanted eye strain due to lens misalignment is known to increase with increasing prescription, a more pronounced benefit in visual performance and comfort may be found by wearing customized frames in subjects with high ametropia compared to the relatively small differences found in this study. Since visual demands, especially for near vision, are becoming increasingly more important in the workplace, there is an increasing demand for improved spectacle comfort, especially for progressive addition lens wearers. However, it is still unknown as to why some wearers adapt very quickly to these lenses while others complain of headaches, swaying effects, and distorted peripheral vision as well as experiencing problems in the workplace. It has already been demonstrated that vergence facility and the rate of phoria adaptation may have potential clinical utility in differentiating which patients may adapt to progressive addition lenses and which ones will have more difficulty.

There are some limitations to this study. First, there is a possible effect of different spherical equivalent values and cornea–vertex distance values measured in the single vision and the progressive addition lens groups on the study outcomes. However, this study has shown that wearing personalized spectacles manufactured with the ideal wearing parameters improved the subjects’ visual performance and overall satisfaction when analyzing the whole cohort. The lack of a statistically significant preference for personalized spectacles when analyzing the SV and PAL groups separately is probably a consequence of the fact that there is little difference between the results when wearing personalized and standard spectacles. Second, the relatively small sample size may make it difficult to determine if a particular outcome is really a true finding, and in some cases, a type I error may occur, especially in the case of multiple comparisons. We recommend further studies with larger sample sizes, which should lead to more reliable conclusions. Another limitation of this study was, that the same aspheric single-vision lens design and progressive addition lens design were used for both the control spectacles and the YUNIKU frames. While this helps to isolate the difference in this study caused by the difference in frames, if the lenses had been customized for the control frames, as is common with many lens manufacturers nowadays, there might have been less of a subjective difference. Finally, it is also possible that if subjects who reported no benefit of personalized progressive addition lenses were to wear progressive addition lenses longer, then these subjects might have eventually shown a preference for personalized progressive addition lenses. However, it is beyond the scope of this study to determine whether subjects who self-reported that they could not choose between personalized and control progressive addition lenses after 1 month would eventually prefer personalized progressive addition lenses if they were given more time to wear them. Further studies on a patient cohort with a higher level of ametropia is suggested to identify the plausible relationship between dioptric power and the probability of non-adaptation to progressive addition lenses.

## 5. Conclusions

This study has shown that wearing personalized spectacles manufactured according to an individual’s facial anatomy with the ideal wearing parameters results in some advantages. Slightly improved visual performance and comfort is noticeable while wearing the personalized spectacles.

## Figures and Tables

**Table 1:** Comparison of the construction characteristics of the personalized and control spectacles.

Criteria	Personalized Spectacles	Control Spectacles
Manufacturing	Personalized frame, produced on demand for each subject	Pre-produced frame based on standard parameters in one fixed size
Measurement and adjustment to the subject’s face topography	Face scan and 3D rendering of the subject’s face Calculation of the ideal lens parameter/position based on prescription, functional requirements, and facial data	Manual adjustment of the frame to the subject’s face The video-centration system visuReal portable is used to measure all frame-related wearing parameters
Definition of frame size and position	The parametric frame model is adjusted to the calculated lens position and face topography	Selection of a frame that fits as best as possible
Lens design	Ideal lens-related parameters are considered	Individual frame-wearing parameters are converted into lens-related parameters

**Table 2:** Fitting properties of the personalized and control spectacles. The standard parameters of traditional prescription eyewear for progressive addition lenses (PAL) and single vision lenses (SV) are also indicated.

		Lens Designs					
		PAL			SV		
		Personalized	Control	Standard	Personalized	Control	Standard
**Vertex Distance [mm]**	**CVD**	13.82 ± 2.51			13.76 ± 3.11		
	**FCD**		16.25 ± 3.77	12.30		14.91 ± 3.07	12.30
**Pantoscopic** **Angle [°]**	**PA**	8.75 ± 2.38			4.04 ± 2.78		
	**WPA**		8.62 ± 4.27	8.30		8.83 ± 2.87	8.30
**Face Form** **Angle [°]**	**LFFA**	1.14 ± 0.45			1.24 ± 0.24		
	**FFFA**		1.75 ± 1.40	4.40		1.31 ± 0.71	4.40

Note: CVD: cornea–vertex distance, FCD: frame–cornea distance, PA: pantoscopic angle, WPA: wearers’ pantoscopic angle, LFFA: lens face form angle, and FFFA: frame face form angle. Data: mean ± standard deviation.

**Table 3:** Scores of the final comparison questionnaires in the single vision (SV) and progressive addition lens (PAL) groups.

Question	SV Group (*n* = 30)			PAL Group (*n* = 30)			All spectacles (*n* = 60)		
	Personalized	Control	*p*	Personalized	Control	*p*	Personalized	Control	*p*
How comfortable are the spectacles in general? (1–5)	4.03 ± 0.93	3.67 ± 1.37	0.30	4.30 ± 0.92	4.03 ± 0.81	0.11	4.17 ± 0.92	3.85 ± 1.13	0.13
How easily and quickly could you adapt to them? (1–5)	3.87 ± 1.14	3.97 ± 1.40	0.71	4.07 ± 1.01	3.80 ± 1.21	0.33	3.97 ± 1.07	3.88 ± 1.30	0.86
How comfortable are the spectacles on your nose? (1–5)	3.77 ± 1.25	3.47 ± 1.43	0.44	4.17 ± 1.05	4.10 ± 0.96	0.34	3.97 ± 1.16	3.78 ± 1.25	0.37
How comfortable are the temples? (1–5)	4.30 ± 1.09	3.73 ± 1.44	0.11	4.27 ± 0.79	4.17 ± 0.79	0.35	4.28 ± 0.94	3.95 ± 1.17	0.10
How satisfied are you with your vision in general? (1–5)	4.77 ± 0.50	4.50 ± 0.97	0.19	4.37 ± 0.89	4.17 ± 1.18	0.36	4.57 ± 0.74	4.33 ± 1.08	0.15
How satisfied are you with the visual field? (1–5)	4.47 ± 0.68	4.33 ± 1.06	0.59	4.30 ± 0.99	4.13 ± 1.04	0.37	4.38 ± 0.85	4.23 ± 1.05	0.28
Did you experience a swaying feeling when wearing the glasses? (1–5)	4.40 ± 0.93	4.23 ± 1.17	0.46	4.27 ± 1.01	3.87 ± 1.20	0.38	4.33 ± 0.97	4.05 ± 1.18	0.06
Please rank the spectacles (1–5)	8.00 ± 1.76	7.27 ± 2.72	0.44	7.97 ± 1.81	7.40 ± 2.46	0.39	7.98 ± 1.80	7.33 ± 2.57	0.18
Which pair of spectacles would you like to keep?	17	13	0.29	20	10	0.40	37	23	0.04
OVERALL SCORE	37.6 ± 6.17	35.17 ± 9.79	0.52	37.73 ± 6.09	35.57 ± 7.55	0.41	37.67 ± 6.08	35.37 ± 8.67	0.17

Note: Patient satisfaction was assessed linearly on a 1–5-point scale, with 5 indicating the best quality of vision or comfort and 1 indicating the worst. *p*: Wilcoxon signed-rank test and binomial test with confidence intervals. Data: mean ± standard deviation.

## Data Availability

Data are available from the authors upon reasonable request.

## Appendix A

**Table A1:** Inclusion and exclusion criteria for the study participants.

Inclusion Criteria	1. Age: - Single vision design (SV group): 18–44 years - Progressive addition lens design (PAL group): 45–65 years 2. The subjects should be experienced wearers wearing SV/PAL designs during the last six months. 3. Visual Acuity: distance and near monocular ≥ 1.0 (≤0.0 logMAR) 4. Normal binocular vision 5. Ametropia: - Spherical power: −6.00–+6.00 D - Cylindrical power: ≤ 2.50 D - Difference in power (spherical equivalent) between TE eyes: ≤ 2.00 D
Exclusion Criteria	1. First prescription for progressive addition lenses 2. The prescription varies from the previous prescription by more than 0.75 D in the spherical equivalent or in the cylinder axis by more than 15° in any eye 3. Double vision or prismatic power in current glasses 4. Known ocular disease including strabismus, any pathology, and any eye surgeries that may affect visual acuity 5. The use of systemic or ocular medication that is likely to affect vision 6. Balance problems/vertigo problems 7. Concurrent participation in any other vision-related studies 8. Pregnancy 9. Inadequate fitting of control frames

**Table A2:** First impression questionnaire.

Question
How comfortable are the new spectacles in general? (1–10)
How comfortable are the new spectacles on your nose? (1–10)
How do you find the spectacles’ bridge on your nose?
1: There is a space between the bridge and my nose
2: It presses on my nose
3: It fits perfectly on my nose
How are the temples? (1–10)
How do you find the temples according to your head?
1: They are too loose on my head
2: They press on my head
3: They fit perfectly according to my head
How is the length of the temples?
1: Too long
2: Too short
3: The temple’s length is exactly what I need
How is your vision in general with the new spectacles? (1–10)
How do you find the field of view with the new spectacles in general? (1–10)
Do you notice any distortion of images or blurring?
1: No, not at all
2: Slightly
Do you experience any swaying feelings?
1: No, not at all
2: Slightly
OVERALL SCORE

Note: Patient satisfaction was assessed linearly on a 1–10-point scale, with 10 indicating the best quality of vision or comfort and 1 indicating the worst.

**Table A3:** Self-evaluation questionnaire.

Question
How comfortable is this pair of spectacles in general? (1–5)
How easily and quickly could you adapt to this pair of spectacles? (1–5)
Spectacles slide down from the nose (1–5)
Lenses steam up while wearing them (1–5)
How comfortable was this pair on your nose during the two weeks of wearing them? (1–5)
How comfortable were the temples during the two weeks of wearing this pair? (1–5)
How satisfied are you with your vision/visual acuity using this pair looking:
At far distances (>4–6 m: TV…)? (1–5)
At intermediate distances (≈60 cm–1 m: computer work) (1–5)
Doing your near work (≈30–50 cm: reading...) (1–5)
How satisfied are you with the visual field using this pair:
At far distances (>4–6 m: TV…)? (1–5)
At intermediate distances (≈60 cm -1 m: computer work) (1–5)
Doing your near work (≈30–50 cm: reading...) (1–5)
Did you experience any swaying feeling using this pair? (1–5)
How satisfied are you with this eyewear in general? (1–5)
OVERALL SCORE

Note: Patient satisfaction was assessed linearly on a 1–5-point scale, with 5 indicating the best quality of vision or comfort and 1 indicating the worst.

**Table A4:** Final comparison questionnaire.

Question
How comfortable are the spectacles in general? (1–5)
How easily and quickly could you adapt to them? (1–5)
How comfortable are the spectacles on your nose? (1–5)
How comfortable are the temples? (1–5)
How satisfied are you with your vision in general? (1–5)
How satisfied are you with the visual field? (1–5)
Did you experience a swaying feeling when wearing the glasses? (1–5)
Please rank the spectacles (1–5)
Which pair of spectacles would you like to keep?
OVERALL SCORE

Note: Patient satisfaction was assessed linearly on a 1–5-point scale, with 5 indicating the best quality of vision or comfort and 1 indicating the worst.

**Table A5:** Results of the first impression questionnaire in the single vision (SV) and progressive addition lenses (PAL) groups.

	SV Group (*n* = 30)			PAL Group (*n* = 30)			All spectacles (*n* = 60)		
Question	Personalized	Control	*p*	Personalized	Control	*p*	Personalized	Control	*p*
How comfortable are new spectacles in general? (1–10)	8.70 ± 1.23	7.46 ± 2.55	0.03	8.40 ± 1.59	8.43 ± 1.53	0.54	8.55 ± 1.42	7.95 ± 2.26	0.19
How comfortable are the new spectacles on your nose? (1–10)	8.23 ± 1.92	7.30 ± 2.84	0.20	8.50 ± 1.19	8.87 ± 1.04	0.14	8.37 ± 1.59	8.08 ± 2.26	0.94
How do you find the spectacles’ bridge on your nose									
1: There is a space between the bridge and my nose	2	8	0.65	5	3	0.31	7	11	0.54
2: It presses on my nose	5	3		1	4		6	7	
3: It fits perfectly on my nose	23	19		24	23		47	42	
How are the temples? (1–10)	8.53 ± 1.66	8. 20 ± 2.19	0.58	8.83 ± 1.08	8.53 ± 1.33	.27	8.68 ± 1.40	8.37 ± 1.80	.31
How do you find the temples according to your head?									
1: They are too loose on my head	8	9	0.96	6	9	0.33	14	18	0.38
2: They press on my head	1	0		0	3		1	3	
3: They fit perfectly according to my head	21	21		24	18		45	39	
How is the length of the temples?									
1: Too long	7	11	0.52	4	6	0.69	11	17	0.29
2: Too short	1	0		2	1		3	1	
3: The temple’s length is exactly what I need	22	19		24	23		46	42	
How is your vision in general with the new spectacles? (1–10)	9.77 ± 0.43	9.37 ± 1.03	0.05	8.47 ± 1.33	7.90 ± 2.41	0.51	9.12 ± 1.18	8.63 ± 1.98	0.19
How do you find the field of view with the new spectacles in general? (1–10)	8. 40 ± 1.96	8.30 ± 1.66	0.59	8.70 ± 1.34	8.40 ± 1.92	0.52	8.55 ± 1.67	8.35 ± 1.78	0.34
Do you notice any distortion of images or blurring?									
1: No, not at all	27	24	0.28	23	20	0.39	50	44	0.18
2: Slightly	3	6		7	10		10	16	
Do you experience any swaying feelings?									
1: No, not at all	24	22	0.54	17	19	0.59	41	41	0.99
2: Slightly	6	8		13	11		19	19	
OVERALL SCORE	43.63 ± 4.73	40.63 ± 8.32	0.20	42.90 ± 4.37	42.13 ± 5.30	0.95	43.27 ± 4.52	41.38 ± 6.96	0.30

Note: Patient satisfaction was assessed linearly on a 1–10-point scale, with 10 indicating the best quality of vision or comfort and 1 indicating the worst. SV: single vision; PAL: progressive addition lens; *p*: Wilcoxon signed-rank test and Chi-squared test for the 2 × 2 or 3 × 2 contingency tables. Data: mean ± standard deviation.

**Table A6:** Scores of the self-evaluation questionnaires in the single vision (SV) and progressive addition lenses (PAL) groups.

	SV Group (*n* = 30)			PAL Group (*n* = 30)			All spectacles (*n* = 60)		
Question	Personalized	Control	*p*	Personalized	Control	*p*	Personalized	Control	*p*
How comfortable is this pair of spectacles in general? (1–5)	4.13 ± 1.01	3.73 ± 1.26	0.17	4.13 ± 0.94	3.90 ± 1.06	0.32	4.13 ± 0.96	3.82 ± 1.16	0.09
How easily and quickly could you adapt to this pair of spectacles? (1–5)	4.13 ± 1.01	4.0 ± 1.44	0.76	3.80 ± 1.16	3.63 ± 1.43	0.59	3.97 ± 1.09	3.82 ± 1.43	0.55
Spectacles slide down from the nose (1–5)	3.57 ± 1.22	3.33 ± 1.40	0.51	3.80 ± 1.27	3.93 ± 1.23	0.56	3.68 ± 1.24	3.63 ± 1.34	0.89
Lenses steam up while wearing them (1–5)	4.33 ± 1.03	4.20 ± 1.09	0.58	4.23 ± 0.90	4.53 ± 0.63	0.06	4.28 ± 0.96	4.37 ± 0.90	0.51
How comfortable was this pair on your nose during the two weeks of wearing them? (1–5)	3.70 ± 1.26	3.53 ± 1.55	0.61	4.23 ± 0.77	4.07 ± 1.05	0.33	3.97 ± 1.07	3.80 ± 1.34	0.34
How comfortable were the temples during the two weeks of wearing this pair? (1–5)	4.30 ± 1.02	3.77 ± 1.48	0.19	3.99 ± 0.99	4.17 ± 0.95	0.38	4.13 ± 1.02	3.97 ± 1.25	0.46
How satisfied are you with your vision/visual acuity using this pair looking:									
At far distances (>4–6 m: TV…)? (1–5)	4. 87 ± 0.34	4.70 ± 0.70	0.29	4.43 ± 0.86	3.80 ± 1.21	0.02	4.65 ± 0.68	4.25 ± 1.08	0.01
At intermediate distances (≈60 cm–1 m: computer work) (1–5)	4.83 ± 0.38	4.73 ± 0.58	0.40	4.03 ± 1.22	3.97 ± 1.25	0.96	4.43 ± 0.98	4.35 ± 1.04	0.77
Doing your near work (≈30–50 cm: reading...) (1–5)	4.77 ± 0.73	4.67 ± 0.61	0.35	4.20 ± 1.21	3.60 ± 1.35	0.02	4.48 ± 0.95	4.13 ± 1.17	0.01
How satisfied are you with the visual field using this pair:									
At far distances (>4–6 m: TV…)? (1–5)	4.03 ± 1.07	4.27 ± 1.17	0.16	4.37 ± 0.99	4.00 ± 1.20	0.10	4.20 ± 1.04	4.13 ± 1.17	0.94
At intermediate distances (≈60 cm–1 m: computer work) (1–5)	4.27 ± 0.91	4.47 ± 0.97	0.19	4.20 ± 1.06	4.07 ± 1.20	0.38	4.23 ± 0.98	4.27 ± 1.10	0.75
Doing your near work (≈30–50 cm: reading...) (1–5)	4.57 ± 0.73	4.57 ± 0.94	0.72	4.27 ± 0.94	4.00 ± 1.26	0.19	4.42 ± 0.85	4.28 ± 1.14	0.41
Did you experience any swaying feeling when using this pair? (1–5)	4. 50 ± 0.73	4.20 ± 1.18	0.19	4.30 ± 1.09	3.80 ± 1.15	0.03	4.40 ± 0.92	4.00 ± 1.18	0.02
How satisfied are you with this eyewear in general? (1–5)	4.10 ± 0.96	3.77 ± 1.22	0.25	4.17 ± 0.87	3.70 ± 1.09	0.04	4.13 ± 0.91	3.73 ± 1.15	0.03
OVERALL SCORE	60.10 ± 7.30	57.93 ± 10.50	0.48	58.13 ± 9.25	55.17 ± 11.18	0.21	59.12 ± 8.32	56.55 ± 10.87	0.18

Note: Patient satisfaction was assessed linearly on a 1–5-point scale, with 5 indicating the best quality of vision or comfort and 1 indicating the worst. SV: single vision; PAL: progressive addition lens *p*: Wilcoxon signed-rank test. Data: mean ± standard deviation.

## References

[1] Sheppard A.L., Wolffsohn J.S. (2018). Digital eye strain: Prevalence, measurement and amelioration. BMJ Open Ophthalmol..

[2] Wolffsohn J.S., Davies L.N. (2019). Presbyopia: Effectiveness of correction strategies. Prog. Retin. Eye Res..

[3] Jaschinski W., König M., Mekontso T.M., Ohlendorf A., Welscher M. (2015). Comparison of progressive addition lenses for general purpose and for computer vision: An office field study. Clin. Exp. Optom..

[4] De Lestrange-Anginieur E., Kee C.S. (2021). Optical performance of progressive addition lenses (PALs) with astigmatic prescription. Sci. Rep..

[5] Kee C.-S., Leung T.W., Kan K.-H.B., Lam C.H.-I.B. (2018). Effects of Progressive Addition Lens Wear on Digital Work in Pre-presbyopes. Optom. Vis. Sci..

[6] Meister D.J., Fisher S.W. (2008). Progress in the spectacle correction of presbyopia. Part 1: Design and development of progressive lenses. Clin. Exp. Optom..

[7] Meister D.J., Fisher S.W. (2008). Progress in the spectacle correction of presbyopia. Part 2: Modern progressive lens technologies. Clin. Exp. Optom..

[8] Sheedy J.E. (2004). Progressive addition lenses—Matching the specific lens to patient needs. Optom. J. Am. Optom. Assoc..

[9] Han Y., Ciuffreda K.J., Selenow A., Ali S.R. (2003). Dynamic interactions of eye and head movements when reading with single-vision and progressive lenses in a simulated computer-based environment. Investig. Opthalmology Vis. Sci..

[10] Legras R., Vincent M., Marin G. (2023). Does visual acuity predict visual preference in progressive addition lenses?. J. Optom..

[11] Sullivan C.M., Fowler C.W. (1990). Investigation of progressive addition lens patient tolerance to dispensing anomalies. Ophthalmic Physiol. Opt..

[12] Sheedy J., Hardy R.F., Hayes J.R. (2006). Progressive addition lenses—Measurements and ratings. Optom. J. Am. Optom. Assoc..

[13] Huang C.-Y., Raasch T.W., Yi A.Y., Bullimore M.A. (2013). Comparison of Progressive Addition Lenses by Direct Measurement of Surface Shape. Optom. Vis. Sci..

[14] Barbero S., Portilla J. (2016). The relationship between dioptric power and magnification in progressive addition lenses. Ophthalmic Physiol. Opt..

[15] Rifai K., Wahl S. (2006). Specific eye–head coordination enhances vision in progressive lens wearers. J. Vis..

[16] Hutchings N., Irving E.L., Jung N., Dowling L.M., Wells K.A. (2007). Eye and head movement alterations in naïve progressive addition lens wearers. Ophthalmic Physiol. Opt..

[17] Alvarez T.L., Kim E.H., Granger-Donetti B. (2017). Adaptation to Progressive Additive Lenses: Potential Factors to Consider. Sci. Rep..

[18] Pascual E., Gómez-Pedrero J.A., Alonso J. (2023). Theoretical performance of progressive addition lenses with poorly measured individual parameters. Ophthalmic Physiol. Opt..

[19] Han S.C., Graham A.D., Lin M.C. (2011). Clinical Assessment of a Customized Free-Form Progressive Add Lens Spectacle. Optom. Vis. Sci..

[20] Hoya Corporation White Paper Hoyalux iD MyStyle V+ 2014, 01. https://www.hoyavision.com/contentassets/cd44fd98c9fb469497d6621fe1db16d9/id-mystyle2-whitepaper_full-version_10_21_20.pdf/.

[21] Radner W., Obermayer W., Richter-Mueksch S., Willinger U., Velikay-Parel M., Eisenwort B. (2002). The validity and reliability of short German sentences for measuring reading speed. Graefe’s Arch. Clin. Exp. Ophthalmol..

[22] Stifter E., König F., Lang T., Bauer P., Richter-Müksch S., Velikay-Parel M., Radner W. (2004). Reliability of a standardized reading chart system: Variance component analysis, test-retest and inter-chart reliability. Graefe’s Arch. Clin. Exp. Ophthalmol..

[23] Selenow A., Bauer E.A., Ali S.R., Spencer L.W., Ciuffreda K.J. (2002). Assessing Visual Performance with Progressive Addition Lenses. Optom. Vis. Sci..

[24] Sánchez-Brau M., Domenech-Amigot B., Brocal-Fernández F., Quesada-Rico J.A., Seguí-Crespo M. (2020). Prevalence of Computer Vision Syndrome and Its Relationship with Ergonomic and Individual Factors in Presbyopic VDT Workers Using Progressive Addition Lenses. Int. J. Environ. Res. Public Health.

[25] Garcia-Espinilla O., Gallegos-Cocho I., Sanchez I., Cañadas P., Martin R. (2022). Comparison of physiognomy and frame angle parameters using different devices to prescribe progressive addition lenses. Clin. Exp. Optom..

[26] Garcia-Espinilla O., Gallegos-Cocho I., Sanchez I., Cañadas P., Martin R. (2023). Interdevice agreement in the measurement of physiognomy parameters and frame angles to prescribe progressive addition lenses. Clin. Exp. Optom..

[27] Fontaine N.O., Hanssens J.-M.O., Nguyen M., Bérubé O. (2023). Ordering Eyeglasses Using 3D Head Scan Technology versus Established Online and Storefront Clinic Methods. Optom. Vis. Sci..

[28] Alionte C.G., Ungureanu L.M., Alexandru T.M. (2022). Innovation Process for Optical Face Scanner Used to Customize 3D Printed Spectacles. Materials.

[29] Campomanes A.G.d.A., Meer E., Clarke M., Brodie F.L. (2022). Using a Smartphone 3-Dimensional Surface Imaging Technique to Manufacture Custom 3-Dimensional–Printed Eyeglasses. JAMA Ophthalmol.

[30] Lee L., Burnett A.M., Panos J.G., Paudel P., Keys D., Ansari H.M., Yu M. (2020). 3-D printed spectacles: Potential, challenges and the future. Clin. Exp. Optom..

